# Psychometric Properties of the Violence Exposure Scale in Ecuadorian Adolescents and Its Relationship with Child-to-Parent Violence

**DOI:** 10.3390/children12101343

**Published:** 2025-10-06

**Authors:** Paola Bustos-Benítez, Andrés Ramírez, Javier Herrero Díez, M. Carmen Cano-Lozano

**Affiliations:** 1 Department of Psychology, Fundación Universitaria Konrad Lorenz, Bogotá 110231, Colombia; paolar.bustosb@konradlorenz.edu.co; 2 Grupo de Investigación en Neurociencia Clínica Aplicada (GINCA), Department of Clinical Psychology, Universidad Politécnica Salesiana, Cuenca 010107, Ecuador; 3 Department of Psychology, Universidad de Oviedo, 33003 Oviedo, Spain; herrero@uniovi.es; 4 Department of Psychology, Universidad de Jaen, 23071 Jaen, Spain; mccano@ujaen.es

**Keywords:** psychometric, violence exposure, adolescents, child-to-parent violence

## Abstract

**Introduction:** Exposure to violence is an adverse experience associated with the perpetration of violent future behaviors such as child-to-parent violence. **Objective:** The objectives were to analyze the psychometric properties of the Violence Exposure Scale (VES) in a sample of Ecuadorian adolescents as well as its measurement invariance by sex and age; analyze the differences in exposure to violence across four settings (home, school, street, and TV), in two time frames (last year and childhood), according to sex and age; and analyze the relationship between exposure to violence in the four settings and in both time frames with child-to-parent violence. **Methods:** A cross-sectional study was conducted using a probabilistic sample of 2150 Ecuadorian adolescents (55% female), aged 12 to 18 years (*M* = 14.53; *SD* = 1.55). Participants completed the adapted version of the VES and the Child-to-Parent Violence Questionnaire (CPV-Q). Confirmatory factor analyses, reliability testing, convergent and discriminant validity analyses, and measurement invariance assessments were performed. **Results:** The VES showed excellent model fit in both versions, VES^1^ (last year) and VES^2^ (before age 10), with strong goodness-of-fit indices (VES^1^: *CFI* = 0.988; *RMSEA* = 0.055; VES^2^: *CFI* = 0.994; *RMSEA* = 0.044). Reliability was good (α_o_ and ω_o_ ≤ 0.80; G.6 and *CR* ≤ 0.70). Effect sizes ranged from 0.11 to 0.31 for violence by children toward parents and reached up to 0.83 among the different forms of victimization. **Conclusions:** The adaptation of the VES in Ecuadorian adolescents showed validity and reliability in assessing exposure to violence. Girls were more at risk at home, while boys were more exposed at school and in the community.

## 1. Introduction

Family violence can be defined as any violent act or pattern of behavior perpetrated by a family member or intimate partner that causes or has the potential to cause physical or psychological harm to the victim [1]. The literature has identified five categories of family violence: (1) child abuse, (2) intimate partner violence (IPV), (3) elder abuse, (4) child-to-parent violence (CPV), and (5) sibling violence [1]. Although the nature of the behaviors is different for each category of violence, there are specific associations between the different violent behaviors [2]. Although the nature of the behaviors is different in each of these categories, two dimensions have been proposed that show their interconnectedness: the threat dimension, mainly related to physical and psychological violence, and the deprivation dimension, which includes physical and psychological neglect [2]. 

Childhood exposure to violent events such as physical, emotional or sexual abuse, neglect, and family dysfunction constitutes an adverse experience [3], which is associated with psychological, social, physical and cognitive impairments [4], as well as increased risk of violent behaviors in adolescence. These experiences are not restricted to the family setting, as they can also manifest themselves at school, through bullying and peer victimization, and in the community, through urban violence, armed conflict or structural discrimination [5].

An important part of the literature indicates that exposure to violent situations can generate vicarious victimization, when violent acts directed at others within or outside the family nucleus are witnessed [6], and direct victimization, when there are physical aggressions between parents and children or intimate partner violence in the home [7]. Both forms often coexist and increase vulnerability to developing internalizing behaviors such as anxiety, depression, and eating disorders and externalizing behaviors such as aggression, substance abuse, and criminal behavior [2,8,9,10]. These experiences affect mental health in the short and long term, impair quality of life and constitute one of the strongest predictors of intimate partner violence in adulthood.

Exposure to family violence and the presence of dysfunctional relationships, characterized by persistent conflict and high levels of stress, create an environment conducive to the reproduction of aggressive behaviors. From social learning theory, it is argued that the normalization of violent patterns leads to the modeling of aggressive behaviors, perceived as effective forms of conflict resolution. This dynamic increases the acceptance of violence in intimate relationships and, with it, the risk of both perpetration and victimization [4]. In this framework, exposure to violence is a significant risk factor in the development of child-to-parent violence (FPV), in which children assume the role of aggressors and parents the role of victims [2,8]. Both direct and indirect exposure to violent situations in the home are positively associated with the occurrence of aggressive behavior towards parents [6].

Studies indicate that adolescents who perpetrate violence against their parents tend to have a history of family victimization and a greater perception of social hostility [8]. In this line, it has been identified that many have been victims of family abuse, both direct and indirect [9]. Likewise, it has been found that young people involved in child-to-parent violence have suffered parental abuse and exposure to interparental violence, which evidences the influence of adverse childhood experiences in the reproduction of violent behaviors towards parents [10].

As demonstrated so far, exposure to violence in the home has received the most attention in international research [11,12,13,14,15,16], but little research has been performed on the relationship between exposure to violence experienced in other contexts, such as on the street or through observing violence in technological media (television), and consequently with CVP. It is understood that the environment in which the individual develops is a relevant mediating factor for subsequent violence [10].

However, it is important to point out that to analyze this association between experiences of violence towards children and the relationship with the perpetration of future violent behaviors or with other variables, it is necessary to have standardized tools that facilitate assessment supported by psychometric evidence. It is currently known that there are several methodological limitations that make it difficult to assess this issue [16,17,18,19,20,21,22,23,24]. This is despite various strategies that the literature has identified to improve measurement strategies [25].

In a recent systematic review [16], it was identified that there are 10 measures that focus on children’s exposure to violence, but it left out instruments such as the Violence Exposure Scale [26], which is a tool widely used in Spanish-speaking countries to assess exposure to violence in four settings, so this review leaves out relevant literature in this field and leads to a bias of excluding important information for this area of study, focusing instead on instruments from specific contexts and high-income settings.

Most studies on exposure to violence (EV) and child-to-parent violence (CPV) have been conducted in high-income countries such as the United States, the United Kingdom, Canada, and Spain [16,27]. As a result, global evidence on the impact of EV on CPV is extremely limited and largely confined to specific sociocultural contexts [28]. In this sense, the most widely used questionnaire to analyze the relationship between EV and CPV is the Violence Exposure Scale (VES) [7,9,22,23,29,30,31]. This scale was developed for a Spanish-speaking population and has demonstrated strong evidence of its validity and adequate psychometric properties [26], including the results of the Colombian validation. However, to our knowledge, this has not been adapted or validated for the Ecuadorian population.

For this reason, this study aims to provide a standardized instrument that allows for the reliable assessment of exposure to violence at home, at school, in the street, and on TV. To this end, we have set the following objectives: (1) to analyze the psychometric properties of the Violence Exposure Scale (VES) in a sample of Ecuadorian adolescents as well as its measurement invariance by sex and age; (2) to analyze the differences in exposure to violence across four settings (home, school, street, and TV), in two time frames (last year and childhood), according to sex and age; and (3) to analyze the relationship between exposure to violence in the four settings and in both time frames with child-to-parent violence.

For this study, the following hypotheses were proposed: (1) The VES is expected to have strong psychometric properties, as well as evidence of validity and reliability for assessing exposure to violence, and to show invariance across genders and age groups. (2) Significant differences in levels of victimization are expected based on gender and age, considering the setting and type of violence. (3) Exposure to violence in the four settings and in both time periods is expected to be significantly associated with children’s violence toward parents.

## 2. Materials and Methods

### 2.1. Participants

The sample consisted of 2150 Ecuadorian adolescents (55% female, 45% male) between 12 and 18 years of age (*M* = 14.53, *SD* = 1.55); 998 (46%) lived in Azuay, 602 (28%) in Cañar, and 550 (26%) in Carchi. The inclusion criteria were adolescents between the ages of 12 and 18 residing in the three provinces of Ecuador (Azuay, Cañar, and Carchi), enrolled in an educational institution, and who agreed to participate in the study through informed consent. The exclusion criteria were adolescents who were using psychoactive substances or who had a disability.

### 2.2. Measures

The Violence Exposure Scale (VES) [26]: This questionnaire consists of 21 items and assesses exposure to violence (direct and vicarious victimization) in four contexts: (1) home, (2) school, (3) street, and (4) TV. In the version adapted to Ecuador, a differentiation based on time frame was included, that is, for the last year (VES^1^) and for childhood/before 10 years of age (VES^2^). This instrument uses a 5-point Likert-type scale (0 = *never* to 4 = *every day*). The reliability of the VES factors in the initial study ranged from α of 0.71 to 0.80 [26].

The Child-to-Parent Violence Questionnaire (CPV-Q) adolescents’ version was initially developed by Contreras and collaborators [32] and later adapted to the Ecuadorian population by [33]. It evaluates the four typologies of CPV: (1) psychological CPV, (2) physical CPV, (3) financial CPV, and (4) behavior of control/domain. The temporality of the behaviors was measured for the last 12 months using a 5-point Likert-type scale (0 = *never* to 4 = *very often/six times or more*). The questions were answered separately for the fathers and mothers. The reliability of the father scale was ω = 0.82, and for the mother it was ω = 0.77.

### 2.3. Procedure

While the questionnaire was initially designed for a Spanish-speaking audience, a linguistic evaluation was performed by native experts in psychometrics and violence to ensure the items were suitable for the Ecuadorian context. Additionally, a pilot study was carried out with a small group of adolescents to analyze the language used, confirming that the cultural interpretation aligned with the objectives of each factor and the overall questionnaire. These steps adhered to the guidelines of the International Test Commission [34].

Following this, approval was secured from Ecuador’s Ministry of Education as well as from the chosen institutions across various provinces, including Azuay, Cañar, and Carchi. The selection of both schools and participants was performed using simple random sampling. Subsequently, individuals aged 12 to 18 were invited to participate, and they were provided with a paper consent form to take home for their parents or legal guardian to sign. Consent was obtained from both the parents and the children involved.

Participation in the study was both voluntary and anonymous, conducted in groups within the classrooms of educational institutions. Adolescents were instructed to complete the paper-and-pencil instruments in a guided manner to minimize variations in response times and prevent external distractions like loss of focus. The individuals overseeing the process were researchers who had received specific training for administering these instruments. Participants remained entirely anonymous, and no incentives were offered for taking part in the study.

### 2.4. Data Analysis

Data analyses were performed using R version 4.4.2 [35]. Descriptive statistics (means and standard deviations) were calculated to summarize the participants’ characteristics. To achieve our first objective, we conducted a confirmatory factor analysis (CFA) using the *Lavvan* statistical package [36] for VES^1^ (last year) and VES^2^ (childhood/before the age of 10). These two-time frames are an original adaptation of this study to differentiate between EV before the age of 10 years and during the last year. The items were treated as ordinal data, and because the data did not present non-normality, the diagonal weighted least squares method (DWLS) was used [37], which is based on a polychoric correlation matrix and asymptotic covariance matrix [38]. The classical psychometric fit criteria used were RMSEA ≤ 0.05, SRMR ≤ 0.080, CFI ≥ 0.95, and TLI ≥ 0.95 [37], as widely referenced [38].

Reliability was calculated using Cronbach’s alpha, McDonald’s omega, and composite reliability (CR) coefficients [38,39]. The acceptable reliability criterion was ≥ 0.65 [39], and the use of the omega coefficient or the CR is recommended. Subsequently, convergent validity was calculated by means of the mean variance extracted (AVE), using the classic reference criterion (AVE ≥ 0.50) [38] as a criterion, indicating that the factor explains more than 50% of the variance [40]. The discriminant validity between the factors was calculated using the heterotrait–monotrait ratio (HTMT ≤ 0.85) [38,40].

To achieve the second objective, invariance tests of VES^1^ and VES^2^ were performed. Invariance was tested as a function of the sex (male vs. female) of the participants and age group (younger adolescents: 12–14 years; older adolescents: 15–18 years). The invariance was calculated as follows: (a) configural (equal factor structure), (b) metric, (c) scalar, and (d) strict [38,41]. To establish whether the VES was invariant according to sex and age, classical suggestions for establishing comparisons between successive models were as follows: (a) CFI ≤ 0.010; (b) RMSEA ≤ 0.015; and SRMR ≤ 0.025 [38,41].

For the third objective, both sex (male and female) and age (12 to 14 years, and 15 to 18 years) were compared as independent groups. A non-parametric Mann–Whitney U test was conducted to compare the scores of exposures to violence at home, at school, in the street, and on television according to sex and age. This procedure was repeated both for the last year and for childhood (before the age of 10). Additionally, the effect size was calculated using the Rank–Biserial Correlation, which is a standardized value and is suggested to be interpreted according to Cohen’s criteria. Finally, for the fulfillment of the fourth objective, a correlation was calculated using the Spearman coefficient to analyze the relationship between exposure to violence in different settings and CPV.

## 3. Results

### 3.1. Psychometric Analysis

The CFA of VES^1^, which evaluates the EV of the last year, presented an excellent model fit: χ^2^ = 1265.004 (*df* = 168; n = 2150; χ^2^/*df* = 7.52); *p* = 0.000; CFI = 0.988; TLI = 0.985; RMSEA = 0.055, 90% de CI (0.052–0.058), SRMR = 0.050; GFI = 0.991; PGFI = 0.567; PNFI = 0.763; ECVI = 1.322. All factor loadings were high and statistically significant for all items (min = 0.688, max = 0.885, see Table A1 for more details).

Subsequently, the structure of the VES^2^ assessing exposure to violence during childhood was tested and presented an excellent model fit: χ^2^ = 848.716 (*df* = 168; n = 2150; χ^2^/*df* = 5.05); *p* = 0.000; CFI = 0.994; TLI = 0.992; RMSEA = 0.044, 90% de CI (0.041–0.047), SRMR = 0.042; GFI = 0.995; PGFI = 0.569; PNFI = 0.794; ECVI = 1.131. All factor loadings were high and statistically significant for all items (min = 0.743, max = 0.914; λ = ≥ 0.50).

Internal consistency was acceptable in all cases (ω_o_ was > 0.80, 95% CI, and CR > 0.70). In addition, other internal consistency coefficients such as ordinal alpha and G.6 were calculated to provide solid evidence of the reliability of the questionnaire. The convergent validity of the five factors of VES^1,2^ explained more than 50% of the variance (AVE > 0.50). Discriminant validity was safe in all cases (HTMT < 0.85); however, the VV at school during childhood and the VV in street factor are at the limit of discriminant validity (see Table 1).

### 3.2. Invariance Measures

The VES^1^ and VES^2^ invariance tests were analyzed as a function of adolescent sex and age and were tested under four added models of configural, metric, scalar and strict invariance. Both models were found to be fully invariant under all four invariance assumptions (See Table 2).

### 3.3. Differences in Exposure to Violence According to the Sex and Age of the Children

In the last year, differences were observed according to sex. At home, women scored significantly higher than men. At school, men showed higher scores than women in both types of victimization. In the street, differences were found only in direct victimization, with men reporting it more frequently. Regarding age differences, it was found that the older group reported higher scores of vicarious victimization at school. Additionally, vicarious violence experienced in the street was higher for the older group.

In childhood, differences were found according to sex. At home, vicarious victimization was mostly reported by girls. At school, boys reported significantly higher scores than girls in both types of victimization. In the street, boys reported higher scores in direct victimization. With regard to age, at home the older group showed higher scores in both types of victimization, and in the street, the older group had higher scores in vicarious victimization (See Table 3).

### 3.4. Relationship Between Exposure to Violence in Different Contexts and CPV

All correlations between CPV and exposure to violence in different contexts were significant. In the last year, a stronger association was observed between CPV toward the father and vicarious victimization in the home (*r* = 0.291, *p* < 0.001) followed by direct victimization in the home (*r* = 0.278, *p* < 0.001). The following contexts with the closest relation were direct exposure to violence at school (*r* = 0.196, *p* < 0.001) and vicarious violence (*r* = 0.175, *p* < 0.001). Exposure to vicarious violence in the street showed a similar association with exposure to violence on TV. Direct exposure to violence in the street showed the weakest association (*r* = 0.113, *p* < 0.001).

In childhood, exposure to vicarious violence in the home and CPV towards the father was the strongest association (*r* = 0.291, *p* < 0.001), followed by direct victimization (*r* = 0.266, *p* < 0.001). At school, it was found that vicarious victimization showed a greater association (*r* = 0.200, *p* < 0.001), following exposure to violence on TV (*r* = 0.187, *p* < 0.001), vicarious victimization in the street (*r* = 0.177, *p* = 0.001) and direct victimization at school (*r* = 0.170, *p* < 0.001). Exposure to direct violence in the street showed the weakest association (*r* = 0.118, *p* < 0.001).

In the last year, the relationship between direct exposure to violence at home and CPV toward the mother showed the strongest association (*r* = 0.317, *p* < 0.001), followed by vicarious victimization in the home (*r* = 0.294, *p* < 0.001). In the school, it was observed that the association was slightly greater when it came to direct victimization (*r* = 0.210, *p* < 0.001), followed by vicarious violence (*r* = 0.192, *p* < 0.001), and exposure to violence on TV showed a similar association (*r* = 0.192, *p* < 0.001). In the street, the relationship was stronger when it came to vicarious victimization (*r* = 0.154, *p* < 0.001) and the least association was with direct victimization in this context (*r* = 0.119, *p* < 0.001).

In childhood, exposure to direct violence and CPV towards the mother showed the strongest association (*r* = 0.305, *p* < 0.001), followed by vicarious victimization (*r* = 0.283, *p* < 0.001). At school, the association was greater when it came to vicarious victimization (*r* = 0.204, *p* = 0.001), followed by direct violence (*r* = 0.199, *p* < 0.001). The next scenario with the highest exposure was TV (*r* = 0.179, *p* < 0.001). In the street, the association was greater for vicarious victimization (*r* = 0.165, *p* < 0.001), and the weakest association among all contexts was direct victimization in the street (*r* = 0.132, *p* < 0.001) (Table 4 and Table 5).

## 4. Discussion

To our knowledge, the VES has not been adapted to and validated in the Ecuadorian population. Therefore, this is the first study to report psychometric properties and validity evidence in a sample of this population. The second original contribution was that the instrument was adapted to differentiate EV experiences in two-time frames, which will allow us to know in detail the effects of EV over time.

### 4.1. Psychometric Properties of the Exposure to Violence Scale

The first objective of this study was to examine the psychometric properties and validity of the VES. This study adapted the original structure of the VES [26] to evaluate the last years of EV (VES^1^) and during childhood/before the age of 10 years (VES^2^). This original contribution showed that both models have an excellent fit for evaluating VE in four settings: home, school, street and TV. The results showed that the VES^2^ version had better goodness-of-fit indices, in addition to being more parsimonious. The internal consistency of VES^1,2^ was acceptable overall and for each of the factors (α_o_ and ω_o_ ≤ 0.80; G.6 and *CR* ≤ 0.70). Both the model’s goodness-of-fit and internal consistency indices were consistent with other studies that have used this instrument to assess EV in Spanish-speaking populations in other contexts [8,9,22,23].

Previous studies have not analyzed evidence of convergent and discriminant validity. Therefore, this study provides novel information on this type of validity. All the factors of VES^1,2^ explained more than 50% of the variance. Discriminant validity was satisfactory among the factors of the VES^1^ but in the VES^2^ caution should be exercised between the factors of VV at school and VV in the street, since the value of HTMT (<0.85) was slightly higher than the criterion value to assume adequate discriminant validity. These findings should be examined in future studies that delve deeper into the effect and contextual differences between the VV of these two scenarios. So far, this contribution suggests that the patterns of victimization between these two contexts may have a high similarity and effect on those who suffer from violence. Since during adolescence there is a growing need for recognition and status among peers, who are agents of socialization outside the home, such as in peer groups and the community, this increases exposure to violence in social contexts, increasing the likelihood of perpetration and victimization of violent behaviors.

### 4.2. Measures of Invariance by Sex and Age

Another objective of this study was to examine the measurement invariance of the VES^1,2^ instrument based on sex and age. The results obtained show that the scale demonstrates full invariance, meaning that configural, metric, and scalar invariance were confirmed across both groups. From a psychometric perspective, these findings are highly relevant, as they indicate that the factorial structure of the instrument is stable and equivalent regardless of the participants’ group membership.

Achieving full invariance implies that the underlying construct of exposure to violence is measured consistently and without bias in both males and females, as well as across different age groups. Technically, this means that the items are interpreted similarly (configural invariance), the strength of the association between the items and the latent factor is equivalent (metric invariance), and the intercepts of the responses are also comparable (scalar invariance). Therefore, any differences found between groups can be attributed to actual differences in exposure to violence rather than to measurement errors or divergent interpretations [41,42].

This psychometric evidence significantly strengthens the internal validity of the instrument and supports its use in future research aimed at comparing populations by sex or age. As a result, it ensures that VES^1,2^ can be confidently used to conduct meaningful, equitable, and unbiased comparisons in both clinical contexts and in epidemiological or intervention studies.

Moreover, confirming invariance across demographic groups broadens the instrument’s applicability in longitudinal studies, cross-cultural evaluations, and differential analyses, which is essential for the development of public policies and prevention strategies tailored to the characteristics of each group. In this way, VES^1,2^ is established as a psychometrically robust tool for assessing exposure to violence across diverse populations.

### 4.3. Analysis of the Differences in Exposure to Violence in Different Scenarios According to Sex and Age in Children

The third objective was to examine the differences between EV a function of different scenarios, sex and age. The differences found provide new information on the role of sex, which is one of the most discussed moderators, as males and females may be affected differently by violence [43,44]. We found that women reported having experienced greater EV at home; specifically, they experienced greater VV during childhood and greater DV and VV in the last year. To some extent, these findings are consistent with worldwide studies showing that females are at risk of experiencing EV [44,45,46] and that child maltreatment is more prevalent in females, so they may be more vulnerable to the effects of child maltreatment and other adverse experiences [47,48].

Secondly, males experienced greater EV in other contexts such as school and the street; in the latter context, it is mostly present as DV. These findings are in line with other research worldwide, which has found that victims of violence at schools were mostly represented by males [49,50]. Street EV, also called “street violence,” was mostly a DV experience and was more often suffered by males, which is consistent with other studies [43,51]. One possible explanation is that males have riskier lifestyles; therefore, they are more likely to be victims or to participate in activities in which they are exposed to situations where violence is more likely to occur [51].

Finally, no differences were found in EV and TV according to sex, and other studies have also found no differences associated with the use of technology [52,53]. In addition, it is important that future studies explore the role of EV on TV, as some studies suggest that such exposure is a risk factor during the preschool years as it contributes to the learning of aggressive behaviors [54,55].

Age plays an important role, as deviant behaviors peak during adolescence [43]. In general, the older adolescent group (15–18 years) presented higher VV in different contexts: VV at school and in the street during the last year, DV and VV at home during childhood, and VV in the street. One possible explanation is that older adolescents have more time to accumulate EV experiences than younger adolescents do [9]. In addition, as they get older, adolescents tend to have greater independence and more activities outside the home, which can lead to a greater likelihood of experiencing victimization in contexts such as school or the street [43,46]. These findings suggest that victimization increases as the age of adolescents increases.

### 4.4. Analysis of the Relationship Between Exposure to Violence and CPV

The data showed that child-to-parent violence (CPV), both toward mothers and fathers, is strongly associated with prior exposure to violence at home, either during childhood or the last year. Specifically, direct exposure to violence at home demonstrated the strongest association with CPV, particularly in the case of violence directed at the mother. The vicarious exposure to violence at home also showed a significant relationship, slightly more pronounced in cases of CPV toward the father. These findings are consistent with previous research that has established both direct and indirect effects of intrafamilial violence on CPV [12,56]. Similarly, prior studies [10,22,33] have reported that a substantial percentage of adolescents who engage in CPV were recurrent victims of either direct or indirect family maltreatment, reinforcing the link between earlier victimization and aggressive behaviors toward parents.

This supports the idea that the way adolescents experience violence within the family whether directly or indirectly can differentially influence abusive behaviors depending on the parent involved. In the school context, both direct and vicarious victimization were moderately associated with CPV, with slight differences between maternal and paternal targets. CPV toward the father showed a slightly stronger association with recent direct victimization at school, whereas, for the mother, both forms of school victimization had similar effects. This suggests that school violence, though less central in explaining CPV, still warrants further exploration, especially given that other studies have shown that higher exposure to violence at home, bullying, and a lower sense of school belonging increase the likelihood of adolescents externalizing their adverse experiences through disruptive or antisocial behaviors.

Exposure to violent television content showed a weak relationship with CPV, though there was a slightly stronger link with CPV toward the mother in the last year. Finally, street violence, whether witnessed or experienced directly, was weakly associated with CPV. In this context, vicarious exposure appeared slightly more relevant than direct exposure, which showed the weakest association with violence toward both parents.

### 4.5. Limitations and Further Research

This study presented limitations that should be acknowledged when interpreting the results. First, due to its cross-sectional design, causal relationships cannot be established, and it remains uncertain whether sex differences in child-to-parent violence (CPV) would persist or vary over time. Second, important contextual variables such as socioeconomic status, parental marital status, educational level, and household composition were not included in the analyses, which may have influenced the findings. Third, all data were obtained through adolescent self-reports, which may be subject to biases such as social desirability or recall errors. Future research should consider dyadic methodologies that incorporate the perspectives of both adolescents and their parents. Moreover, exposure to violence was assessed only in the context of television, while current forms of victimization also emerge from social media, video games, and online pornography. Future studies should expand the scope of media-related exposure to better capture its impact on CPV. Finally, from a psychometric standpoint, although the instruments used showed adequate internal consistency and factorial validity, the study did not include longitudinal analysis or pretest–posttest designs. Future research should adopt longitudinal methodologies and include pre- and post-intervention assessments to evaluate test–retest reliability, temporal stability, and sensitivity to change.

This study has a significant theoretical limitation, as it is difficult to contrast the findings with existing theories and the Latin American context. Nevertheless, the research continues to contribute to the theoretical and empirical development of contexts of exposure to violence in adolescents and its relationship to child-perpetrated violence against parents (CPV), especially in Latin America, a region with social and cultural characteristics different from those of Europe or the United States, where most of the existing literature originates. Additionally, a relevant limitation lies in the scarce availability of previous research specifically addressing the relationship between exposure to violent content in media, such as television, and child-to-parent violence, as well as the influence of violence witnessed or experienced in public spaces, such as the street. This lack of background studies hinders the comparison and contextualization of findings. Therefore, it is necessary to deepen future research on these contexts of exposure. On the other hand, the results of this study support previous findings that show how experiences of violence during childhood and adolescence vary according to sex, age, and the context in which they occur, influencing the later expression of abusive behavior toward parental figures. In this regard, the need to direct prevention and intervention strategies primarily toward the early stages of adolescent development is emphasized, considering the social, cultural, and structural particularities of each group.

Furthermore, the importance of deepening the analysis of variables that contribute to a comprehensive understanding of family and parental violence dynamics is highlighted. This is especially relevant considering that child-to-parent violence (CPV) tends to occur in family contexts characterized by low levels of cohesion and control [57]. Moreover, since the school environment constitutes a key space for adolescents’ socialization, it is pertinent to examine which school-related factors might be associated with the emergence or maintenance of CPV, such as peer influence, bullying, low academic performance, or involvement in risky behaviors.

Finally, this study has several strengths such as the probabilistic sample design, where the participants were randomly selected, in addition to the fact that they lived in geographically separated provinces. Because Ecuador is a culturally diverse country, this finding provides additional information. The effects of settings such as school, street and TV could not be easily contrasted with previous literature; to our knowledge, this is the first study to study separately the effect of VE in different contexts.

### 4.6. Implications

First, the findings of this study can be applied to a standardized test with robust psychometric properties for psychologists and other professionals interested in assessing EV in Ecuadorian adolescents. Second, this study suggests that CPV interventions should contemplate that EV at home has a greater effect than EV in other contexts; therefore, prevention strategies should be focused primarily on the family. However, although with less effect, EV in other contexts is an adverse experience that contributes to explaining CPV towards both parents; therefore, a second study should be carried out for the school and the community.

In the same way, this is a standardized test with robust psychometric properties, making it a useful tool for psychologists and other professionals interested in assessing exposure to violence (EV) among Ecuadorian adolescents. The scale demonstrated a clear and replicable factorial structure, evidenced by satisfactory fit indices in the confirmatory factor analysis (CFI > 0.95; RMSEA < 0.06), as well as adequate levels of internal reliability in its dimensions (α and ω > 0.80). Furthermore, factorial invariance by sex and age was confirmed, ensuring that scores are comparable across groups, a fundamental aspect for studies aiming to identify population differences. Convergent and discriminant validity were also supported through significant correlations with other theoretically related measures. However, it is recommended that future research conduct longitudinal evaluations and test–retest stability studies to strengthen the evidence on the consistency and sensitivity to change of this tool in intervention or clinical follow-up contexts.

Secondly, this study suggests that CPV interventions should consider that exposure to violence (EV) within the home has a greater impact than EV in other contexts. Therefore, prevention strategies should focus primarily on the family, since its involvement in the therapeutic process is key to achieving more effective intervention [13]. On the other hand, although cognitive–behavioral interventions targeting criminogenic needs are effective in some cases, these approaches should be complemented with clinical interventions for youth exhibiting higher levels of dangerousness or relevant clinical symptoms. It is important to consider factors such as mental health, trauma, environmental precariousness, and substance use [58]. Nonetheless, although with less impact, EV in other contexts constitutes adverse experiences that also contribute to explaining CPV toward both parents. Therefore, a secondary effort should be directed at the school and the community.

## 5. Conclusions

This study validated the adapted version of the Violence Exposure Scale (VES) in Ecuadorian adolescents, confirming its reliability and usefulness in different contexts: home, school, street, and television. The scale showed adequate psychometric properties and maintains its validity regardless of gender or age, allowing for the comparison of results between groups and the design of specific intervention strategies from different contexts in which adolescents may interact, such as school, psychotherapeutic, or medical settings.

The results also revealed significant differences in patterns of victimization based on gender, age, and context. In terms of exposure to violence, girls reported higher levels of vicarious victimization at home, while boys experienced higher levels of both direct and vicarious victimization at school and on the street. In addition, older adolescents tended to report more experiences of violence, both at home and on the street, which may reflect a cumulative effect related to greater independence and increased time spent in various environments.

When associating this variable with child-to-parent violence (CPV), the findings show that violence in the home, especially direct violence, is mainly related to aggressive behavior toward the mother, while indirect violence is more closely linked to violence toward the father. Likewise, exposure to school violence shows a significant influence, in contrast to violence on the street or on television, whose relationship is weaker. Overall, the study confirms the relationship between violence experienced or witnessed, particularly at home, and CPV, highlighting the central role of the family environment in the development of these behaviors. In conclusion, the results highlight the importance of the family environment as a key factor and the need to consider variables such as gender, age, and context of exposure when designing preventive strategies and clinical interventions. This research constitutes a significant advance in the study of violence in Latin American contexts and reinforces the relevance of comprehensive, culturally sensitive, and evidence-based approaches to addressing domestic violence in adolescence.

## Figures and Tables

**Table 1:** Reliability, convergent and discriminant validity of the VES.

	α_o_	G.6		ω_o_		CR		AVE		HTMT						
										DV Home	VV Home	DV School	VV School	DV Street	VV Street	TV
VES^1^ (Last year)																
DV home	0.86	0.81	0.87		0.811		0.679		-							
VV home	0.89	0.84	0.89		0.830		0.729		0.849		-					
DV school	0.80	0.75	0.82		0.739		0.591		0.465		0.385	-				
VV school	0.82	0.77	0.84		0.788		0.618		0.391		0.375	0.803	-			
DV street	0.83	0.77	0.84		0.725		0.620		0.281		0.213	0.768	0.429	-		
VV street	0.80	0.73	0.81		0.764		0.577		0.310		0.275	0.509	0.825	0.602	-	
TV	0.87	0.82	0.87		0.835		0.689		0.380		0.342	0.392	0.608	0.349	0.725	1
VES^2^ Childhood (before 10 years of age)																
DV home	0.89	0.85	0.90		0.847		0.747		-							
VV home	0.91	0.87	0.91		0.850		0.768		0.848		-					
DV school	0.86	0.81	0.87		0.792		0.685		0.428		0.368	-				
VV school	0.85	0.80	0.86		0.799		0.664		0.455		0.422	0.842	-			
DV street	0.84	0.79	0.85		0.728		0.650		0.295		0.283	0.711	0.550	-		
VV street	0.82	0.75	0.82		0.759		0.605		0.419		0.376	0.543	0.888	0.635	-	
TV	0.88	0.83	0.88		0.837		0.711		0.435		0.392	0.447	0.662	0.400	0.803	-

Note. α_o_ = Cronbach’s alpha ordinal; G.6 = reliability coefficient G6; ω_o_ = McDonald’s omega ordinal; CR: reliability coefficient; AVE = average variance extracted; HTMT = heterotrait–monotrait ratio; DV = direct victimization; VV = vicarious victimization.

**Table 2:** Invariance of the violence exposure scale by sex and age of the children.

	*x*^2^/*df*	CFI	TLI	RMSEA	SRMR	ΔCFI	ΔRMSEA	ΔSRMR
VES^1^ sex								
M_C_	667.532/336	0.990	0.987	0.030 (0.027–0.034)	0.045			
M_C_⟷M_M_	703.139/350	0.989	0.987	0.031 (0.027–0.034)	0.046	0.001	0.001	0.001
M_M_⟷M_E_	716.808/364	0.989	0.987	0.030 (0.027–0.033)	0.047	0.000	0.001	0.001
M_E_⟷M_S_	741.726/385	0.989	0.988	0.029 (0.026–0.033)	0.049	0.000	0.001	0.002
VES^1^ age								
M_C_	664.912/336	0.990	0.987	0.030 (0.027–0.034)	0.044			
M_C_⟷M_M_	683.051/350	0.990	0.988	0.030 (0.026–0.033)	0.045	0.000	0.000	0.001
M_M_⟷M_E_	706.302/364	0.989	0.988	0.030 (0.026–0.033)	0.045	0.001	0.000	0.000
M_E_⟷M_S_	720.278/385	0.990	0.989	0.028 (0.025–0.032)	0.046	0.001	0.002	0.001
VES^2^ sex								
M_C_	402.548/336	0.998	0.997	0.014 (0.008–0.018)	0.037			
M_C_⟷M_M_	452.612/350	0.997	0.996	0.017 (0.012–0.021)	0.039	0.001	0.003	0.002
M_M_⟷M_E_	464.458/364	0.997	0.996	0.016 (0.011–0.020)	0.040	0.000	0.000	0.001
M_E_⟷M_S_	490.104/385	0.997	0.997	0.016 (0.011–0.020)	0.043	0.000	0.000	0.003
VES^2^ age								
M_C_	414.767/336	0.998	0.997	0.015 (0.009–0.019)	0.037			
M_C_⟷M_M_	459.565/350	0.997	0.996	0.017 (0.013–0.021)	0.039	0.001	0.002	0.002
M_M_⟷M_E_	463.928/364	0.997	0.997	0.016 (0.011–0.020)	0.039	0.000	0.001	0.000
M_E_⟷M_S_	475.579/195	0.997	0.997	0.015 (0.010–0.019)	0.041	0.000	0.001	0.002

Note. M_C_ = configural invariance; M_M_ = metric invariance; M_E_ = scalar invariance; M_S_ = strict invariance; ΔRMSEA < 0.015; ΔCFI < 0.010.

**Table 3:** Differences in average violence exposure scores according to the sex and age of the children.

		Last Year				Childhood			
	G	*M (SD)*	*U*	*p*	*R-B C*	*M (SD)*	*U*	*p*	*R-B C*
**Sex**									
DV home	M	3.508 (3.748)	525,679,000	0.001	−0.080	3.764 (4.048)	552,751,000	0.185	−0.032
	F	4.029 (3.949)				4.012 (4.153)			
VV home	M	2.660 (3.468)	506,731,000	<0.001	−0.113	3.213 (4.005)	534,784,500	0.008	−0.064
	F	3.351 (3.826)				3.591 (4.107)			
DV school	M	1.845 (2.231)	655,853,500	<0.001	0.148	1.607 (2.340)	635,716,500	<0.001	0.113
	F	1.317 (1.951)				1.156 (2.050)			
VV school	M	3.113 (2.775)	635,804,000	<0.001	0.113	2.261 (2.705)	623,598,000	<0.001	0.092
	F	2.579 (2.600)				1.762 (2.338)			
DV street	M	1.289 (2.059)	651,423,500	<0.001	0.140	0.985 (1.868)	630,110,000	<0.001	0.104
	F	0.781 (1.537)				0.603 (1.402)			
VV street	M	3.446 (2.920)	597,351,500	0.066	0.046	2.396 (2.731)	583,585,500	0.376	0.021
	F	3.156 (2.681)				2.210 (2.497)			
TV	M	4.427 (3.600)	560,053,500	0.429	−0.020	3.197 (3.405)	561,608,000	0.767	0.007
	F	4.458 (3.271)				3.045 (3.179)			
**Age**									
DV home	G1	3.904 (3.859)	598,903,500	0.136	0.037	3.651 (3.987)	540,168,000	0.008	−0.065
	G2	3.690 (3.876)				4.147 (4.210)			
VV home	G1	2.948 (3.618)	567,886,500	0.476	−0.017	3.115 (3.923)	527,200,500	<0.001	−0.088
	G2	3.135 (3.750)				3.725 (4.180)			
DV school	G1	1.629 (2.153)	599,390,000	0.114	0.037	1.396 (2.261)	587,961,000	0.436	0.018
	G2	1.478 (2.038)				1.319 (2.129)			
VV school	G1	2.730 (2.735)	549,366,000	0.045	−0.049	1.924 (2.548)	555,492,000	0.106	−0.039
	G2	2.904 (2.647)				2.045 (2.492)			
DV street	G1	0.934 (1.659)	567,774,500	0.425	−0.017	0.775 (1.590)	579,530,500	0.843	0.004
	G2	1.081 (1.939)				0.773 (1.683)			
VV street	G1	3.020 (2.708)	515,865,500	<0.001	−0.107	2.157 (2.511)	547,199,000	0.028	−0.053
	G2	3.548 (2.853)				2.428 (2.689)			
TV	G1	4.306 (3.356)	553,473,000	0.089	−0.042	3.034 (3.191)	557,044,000	0.621	−0.012
	G2	4.581 (3.480)				3.190 (3.369)			

Note. M = male; F = female; G1 = 12 to 14 years; G2 = 15 to 18 years; R-B C = Rank–Biserial Correlation; DV = direct victimization; VV = vicarious victimization; U = Mann–Whitney U test; Childhood = before 10 years of age.

**Table 4:** Correlations of child-to-parent violence with exposure to violence in different childhood settings.

Variable		CPV	DVA	VVA	DVB	VVB	DVC	VVC	TV
1. CPV	Spearman’s rho	—	0.317	0.294	0.210	0.192	0.119	0.154	0.192
	*p*-value	—	<0.001	<0.001	<0.001	<0.001	<0.001	<0.001	<0.001
	Effect size (Fisher’s z)	—	0.328	0.303	0.213	0.194	0.119	0.155	0.195
	SE effect size	—	0.024	0.023	0.023	0.023	0.023	0.023	0.023
2. DVA	rho	0.278	—	0.670	0.325	0.281	0.189	0.237	0.311
	*p*-value	<0.001	—	<0.001	<0.001	<0.001	<0.001	<0.001	<0.001
	Effect size (Fisher’s z)	0.285	—	0.810	0.337	0.288	0.191	0.242	0.322
	SE effect size	0.026	—	0.024	0.024	0.023	0.023	0.023	0.024
3. VVA	rho	0.291	0.666	—	0.291	0.362	0.208	0.332	0.347
	*p*-value	<0.001	<0.001	—	<0.001	<0.001	<0.001	<0.001	<0.001
	Effect size (Fisher’s z)	0.300	0.803	—	0.300	0.379	0.211	0.345	0.362
	SE effect size	0.026	0.027	—	0.026	0.026	0.025	0.026	0.026
4. DVB	rho	0.196	0.319	0.259	—	0.564	0.502	0.395	0.328
	*p*-value	<0.001	<0.001	<0.001	—	<0.001	<0.001	<0.001	<0.001
	Effect size (Fisher’s z)	0.199	0.331	0.265	—	0.639	0.553	0.418	0.341
	SE effect size	0.025	0.026	0.026	—	0.024	0.024	0.024	0.024
5. VVB	rho	0.175	0.276	0.277	0.557	—	0.285	0.643	0.500
	*p*-value	<0.001	<0.001	<0.001	<0.001	—	<0.001	<0.001	<0.001
	Effect size (Fisher’s z)	0.177	0.283	0.285	0.629	—	0.293	0.763	0.549
	SE effect size	0.025	0.026	0.026	0.026	—	0.023	0.024	0.024
6. DVC	rho	0.113	0.164	0.141	0.474	0.247	—	0.403	0.248
	*p*-value	<0.001	<0.001	<0.001	<0.001	<0.001	—	<0.001	<0.001
	Effect size (Fisher’s z)	0.114	0.165	0.142	0.516	0.252	—	0.427	0.254
	SE effect size	0.025	0.025	0.025	0.026	0.026	—	0.024	0.023
7. VVC	rho	0.169	0.237	0.232	0.376	0.645	0.370	—	0.574
	*p*-value	<0.001	<0.001	<0.001	<0.001	<0.001	<0.001	—	<0.001
	Effect size (Fisher’s z)	0.171	0.242	0.237	0.395	0.767	0.389	—	0.654
	SE effect size	0.025	0.026	0.026	0.026	0.027	0.026	—	0.024
8. TV	rho	0.169	0.287	0.268	0.302	0.494	0.205	0.583	—
	*p*-value	<0.001	<0.001	<0.001	<0.001	<0.001	<0.001	<0.001	—
	Effect size (Fisher’s z)	0.170	0.296	0.275	0.312	0.541	0.208	0.667	—
	SE effect size	0.025	0.026	0.026	0.026	0.026	0.025	0.026	—

Note. CPV: child-to-parent violence (the lower triangle represents the father, while the upper triangle represents the mother). DVA = direct victimization at home; VVA = vicarious victimization at home; DVB = direct victimization at school; VVB = vicarious victimization at school; DVC = direct victimization on the street; VVC = vicarious victimization on the street; *rho = Spearman’s rho.*

**Table 5:** Correlations of child-to-parent violence with exposure to violence in different settings in the last year.

Variable		CPV	DVA2	VVA2	DVB2	VVB2	DVC2	VVC2	TV2
1. CPV	rho	—	0.305	0.283	0.199	0.204	0.132	0.165	0.179
	*p*-value	—	<0.001	<0.001	<0.001	<0.001	<0.001	<0.001	<0.001
	Effect size (Fisher’s z)	—	0.315	0.291	0.201	0.206	0.133	0.167	0.181
	SE effect size	—	0.023	0.023	0.023	0.023	0.023	0.023	0.023
2. DVA	rho	0.266	—	0.680	0.354	0.377	0.234	0.352	0.379
	*p*-value	<0.001	—	<0.001	<0.001	<0.001	<0.001	<0.001	<0.001
	Effect size (Fisher’s z)	0.273	—	0.830	0.370	0.396	0.238	0.367	0.399
	SE effect size	0.026	—	0.024	0.024	0.024	0.023	0.024	0.024
3. VVA	rho	0.291	0.685	—	0.276	0.346	0.208	0.305	0.326
	*p*-value	<0.001	<0.001	—	<0.001	<0.001	<0.001	<0.001	<0.001
	Effect size (Fisher’s z)	0.300	0.838	—	0.283	0.361	0.211	0.315	0.338
	SE effect size	0.026	0.027	—	0.023	0.024	0.023	0.023	0.024
4. DVB	rho	0.170	0.342	0.291	—	0.601	0.511	0.431	0.373
	*p*-value	<0.001	<0.001	<0.001	—	<0.001	<0.001	<0.001	<0.001
	Effect size (Fisher’s z)	0.172	0.356	0.300	—	0.695	0.564	0.462	0.391
	SE effect size	0.025	0.026	0.026	—	0.024	0.024	0.024	0.024
5. VVB	rho	0.200	0.372	0.362	0.599	—	0.347	0.668	0.542
	*p*-value	<0.001	<0.001	<0.001	<0.001	—	<0.001	<0.001	<0.001
	Effect size (Fisher’s z)	0.203	0.391	0.379	0.691	—	0.362	0.806	0.607
	SE effect size	0.025	0.026	0.026	0.026	—	0.024	0.024	0.024
6. DVC2	rho	0.118	0.215	0.208	0.497	0.338	—	0.431	0.284
	*p*-value	<0.001	<0.001	<0.001	<0.001	<0.001	—	<0.001	<0.001
	Effect size (Fisher’s z)	0.119	0.219	0.211	0.545	0.352	—	0.461	0.292
	SE effect size	0.025	0.026	0.025	0.026	0.026	—	0.024	0.024
7. VVC	rho	0.177	0.365	0.332	0.422	0.663	0.431	—	0.620
	*p*-value	<0.001	<0.001	<0.001	<0.001	<0.001	<0.001	—	<0.001
	Effect size (Fisher’s z)	0.178	0.383	0.345	0.450	0.799	0.461	—	0.725
	SE effect size	0.025	0.026	0.026	0.026	0.027	0.026	—	0.024
8. TV	rho	0.187	0.381	0.347	0.367	0.541	0.282	0.638	—
	*p*-value	<0.001	<0.001	<0.001	<0.001	<0.001	<0.001	<0.001	—
	Effect size (Fisher’s z)	0.189	0.401	0.362	0.385	0.606	0.290	0.755	—
	SE effect size	0.026	0.026	0.026	0.026	0.026	0.026	0.027	—

Note. CPV: child-to-parent violence (the lower triangle represents the father, while the upper triangle represents the mother). DVA = direct victimization at home; VVA = vicarious victimization at home; DVB = direct victimization at school; VVB = vicarious victimization at school; DVC = direct victimization on the street; VVC = vicarious victimization on the street; *rho = Spearman’s rho*.

## Data Availability

The data presented in this study are available on request from the corresponding author due to ethical approval requirements.

## Appendix A

**Table A1:** Factor loadings.

Factor	Item	VES^1^ (Last Year)				VES^2^ (Childhood/Before 10 Years of Age)			
		λ	Mean (*SD*)	SK	KT	λ	Mean (*SD*)	SK	KT
Home	1A	0.794	0.980 (1.389)	1.181	−0.026	0.824	1.216 (1.556)	0.845	−0.901
	3A	0.885	0.781 (1.323)	1.535	0.911	0.890	0.949 (1.479)	1.224	−0.123
	5A	0.880	1.280 (1.612)	0.782	−1.061	0.914	1.258 (1.636)	0.797	−1.084
	2A	0.778	1.217 (1.478)	0.831	−0.810	0.838	1.448 (1.596)	0.562	−1.293
	4A	0.839	1.229 (1.503)	0.812	−0.873	0.877	1.300 (1.591)	0.725	−1.121
	6A	0.853	1.350 (1.581)	0.667	−1.172	0.877	1.153 (1.557)	0.918	−0.818
School	1B	0.700	0.773 (0.987)	1.209	0.891	0.754	0.610 (0.963)	1.632	2.062
	3B	0.829	0.830 (1.018)	1.092	0.460	0.839	0.616 (0.971)	1.569	1.743
	5B	0.822	1.215 (1.255)	0.693	−0.619	0.848	0.760 (1.084)	1.331	0.845
	2B	0.688	0.317 (0.739)	2.835	8.695	0.782	0.395 (0.835)	2.413	5.675
	4B	0.783	0.392 (0.792)	2.304	5.374	0.840	0.361 (0.794)	2.527	6.371
	6B	0.828	0.844 (1.203)	1.203	0.589	0.858	0.601 (1.011)	1.774	2.452
Street	1C	0.718	1.121 (1.115)	0.667	−0.492	0.743	0.775 (1.056)	1.224	0.591
	3C	0.756	0.926 (1.089)	0.947	−0.063	0.786	0.680 (1.028)	1.406	1.052
	5C	0.803	1.239 (1.224)	0.590	−0.764	0.803	0.839 (1.118)	1.171	0.408
	2C	0.727	0.253 (0.667)	3.143	10.705	0.774	0.221 (0.630)	3.372	12.199
	4C	0.817	0.290 (0.734)	2.949	8.891	0.811	0.214 (0.644)	3.647	14.351
	6C	0.815	0.466 (0.873)	2.037	3.782	0.833	0.339 (0.787)	2.699	7.376
TV	1D	0.790	1.671 (1.309)	0.136	−1.151	0.815	1.168 (1.312)	0.734	−0.754
	3D	0.850	1.335 (1.313)	0.493	−1.034	0.863	0.955 (1.229)	0.999	−0.224
	5D	0.850	1.438 (1.340)	0.405	−1.120	0.851	1.005 (1.270)	0.981	−0.300
